# Transcriptional and post-transcriptional regulation of PenA β-lactamase in acquired *Burkholderia pseudomallei* β-lactam resistance

**DOI:** 10.1038/s41598-018-28843-7

**Published:** 2018-07-13

**Authors:** Sunisa Chirakul, Michael H. Norris, Sirawit Pagdepanichkit, Nawarat Somprasong, Linnell B. Randall, James F. Shirley, Bradley R. Borlee, Olga Lomovskaya, Apichai Tuanyok, Herbert P. Schweizer

**Affiliations:** 1 University of Florida, College of Medicine, Emerging Pathogens Institute, Department of Molecular Genetics and Microbiology, Gainesville, FL 32610 USA; 2 University of Florida, College of Veterinary Medicine, Emerging Pathogens Institute, Department of Infectious Diseases and Immunity, Gainesville, FL 32610 USA; 3 Chulalongkorn University, Faculty of Veterinary Science, Department of Veterinary Public Health, Research Unit in Microbial Food Safety and Antimicrobial Resistance, Bangkok, 10330 Thailand; 4 Colorado State University, College of Veterinary Medicine and Biomedical Sciences, Department of Microbiology, Immunology and Pathology, Fort Collins, CO 80523 USA; 5 The Medicines Company, San Diego, CA 92121 USA; 6 Present Address: Cornell University, Boyd Thompson Institute, Ithaca, NY 14853 USA

## Abstract

Therapy of *Burkholderia pseudomallei* acute infections is largely limited to a few β-lactam antibiotics such as ceftazidime or meropenem. Although relatively rare, resistance emergence during therapy leads to treatment failures with high mortality rates. In the absence of acquired external resistance determinants in *B*. *pseudomallei* emergence of β-lactam resistance is invariably caused by mutational modification of genomically encoded factors. These include the deletion of the ceftazidime target penicillin-binding protein 3 or amino acid changes in the Class A PenA β-lactamase that expand its substrate spectrum, as well as *penA* gene duplication and amplification or its overexpression via transcriptional up-regulation. Evidence is presented that *penA* is co-transcribed with the upstream *nlpD1* gene, that the transcriptional terminator for *nlpD1* serves as a *penA* attenuator and that generation of a new promoter immediately upstream of the terminator/attenuator by a conserved G to A transition leads to anti-termination and thus constitutive PenA expression and extended β-lactam resistance. Further evidence obtained with the extensively β-lactam resistant clinical isolate Bp1651 shows that in addition to PenA overexpression and structural mutations other adaptive mechanisms contribute to intrinsic and acquired *B*. *pseudomallei* β-lactam resistance.

## Introduction

*Burkholderia pseudomallei* is an opportunistic pathogen prevalent in tropical and sub-tropical regions around the world where it causes melioidosis, a multifacteted disease syndrome^1,2^. *B*. *pseudomallei* infections are difficult to treat because of the bacterium’s intrinsic antibiotic resistance^3,4^. Although not yet well studied in *B*. *pseudomallei*, this intrinsic resistance is likely complex and due to an impermeable outer membrane, expression of endogenous resistance factors such as efflux pumps and β-lactamase, as well as biofilm and intracellular lifestyles^5–8^. Unlike most other Gram-negative bacteria, acquired resistance due to horizontal gene transfer has not yet been documented in *B*. *pseudomallei*, but rather is due to expression of chromosomally-encoded resistance determinants. Because β-lactam antibiotics are crucial for acute phase melioidosis therapy, acquired resistance to these antibiotics has been studied in some detail. *B*. *pseudomallei* is intrinsically resistant to many β-lactam antibiotics such as amoxicillin and carbenicillin due to expression of chromosomally-encoded PenA β-lactamase^9^. In combination with clavulanic acid, amoxicillin has clinical utility^4^. Because PenA exhibits only weak cephalosporinase activity, and even weaker carbapenemase activity, ceftazidime (CAZ) and meropenem (MEM) are drugs of choice for treatment of acute *B*. *pseudomallei* infections^4^. However, acquired CAZ resistance (CAZ^r^) by up-regulation of *penA* due to a putative promoter mutation^10–12^, *penA* gene duplication and amplification^13^, and PenA amino acid substitutions that extend the enzyme’s substrate spectrum^10,12,14–16^ have all been shown to occur in clinical isolates as the result of CAZ therapy. Although PenA is the prominent player in *B*. *pseudomallei*’s CAZ^r^, other mechanisms do likely exist but only CAZ^r^ due to penicillin-binding protein 3 target deletion has been shown^17^. We previously described a strain of Australian origin, Bp1651, that exhibited a multidrug resistant phenotype, including resistance to CAZ and imipenem (IPM), and reduced susceptibility to MEM^12^. The data were consistent with the hypothesis that β-lactam resistance in this strain is multifactorial, with *penA* likely being the dominant player, e.g. via synergy between *penA* up-regulation and novel PenA structural mutations that result in high level CAZ and IPM resistance^12^. Synergy leading to CAZ^r^ has been observed before in other clinical isolates^10^. In this study, we build on previous observations and examine the chromosomal organization of *penA* and dominant regulatory features that affect its expression in and contribution to β-lactam resistance of Bp1651 by comparison with the CAZ susceptible (CAZ^s^) prototype strain 1026b.

## Results

### Organization of the *penA* chromosomal region

**Figure 1:**
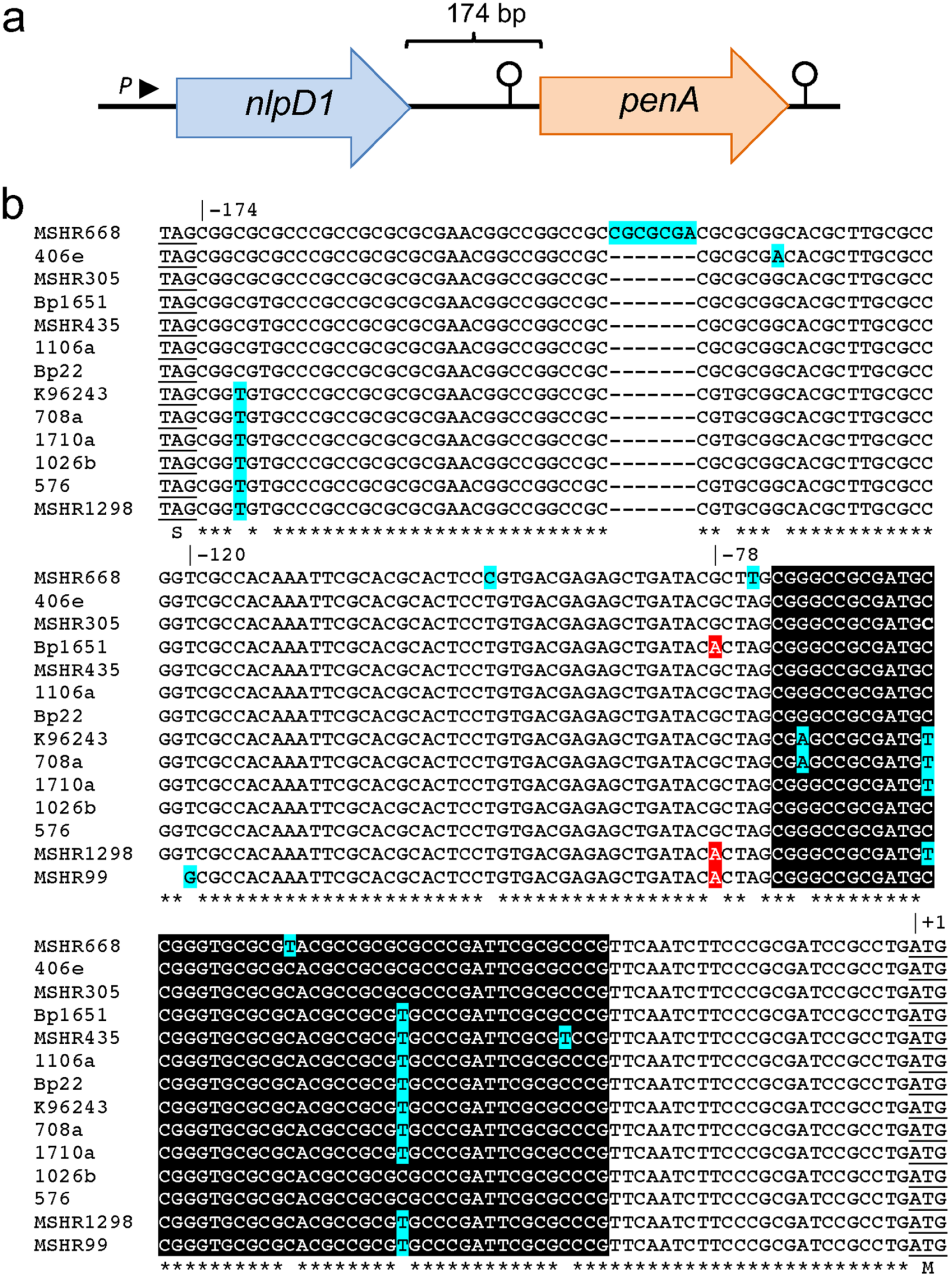
Organization of the *B*. *pseudomallei penA* region on chromosome 2. (**a**) Map of the *penA* region with pertinent features. Wild-type strains contain no recognizable promoter in the 174 bp *penA* upstream region, only a predicted promoter (*P*) upstream of *nlpD1*. This arrangement suggests that *nlpD1* and *penA* form an operon. Lollipop structures indicate proposed transcriptional terminators. The *nlpD1* gene encodes a lipoprotein with homology to *E*. *coli* cell wall hydrolytic amidase activating NlpD. (**b**) Sequence of the *nlpD1*-*penA* intergenic region of selected strains. Of the indicated strains of mostly Australian and Thai origin, only Bp1651, MSHR1298 and MSHR99 are known to be CAZ resistant. All sequences except MSHR99 represent complete *nlpD1-penA* intergenic regions. The publicly available MSHR99 sequence extends to position −120 relative to the first nucleotide of the ATG start codon. Asterisks indicate identical nucleotides and variable nucleotides are shaded in turquoise, including a 7 bp insertion in MSHR668. The A nucleotides present at position −78 in the Bp1651, MSHR1298 and MSHR99 sequences are highlighted in red. (Note: Due to an outdated and mistaken annotation of the *penA* translational start site in several sequenced reference strains some of the literature refers to the G to A transition at position −21A.) Nucleotides located between −73 and −25 forming a proposed transcriptional terminator upstream of *penA* are boxed in black.

**Figure 2:**
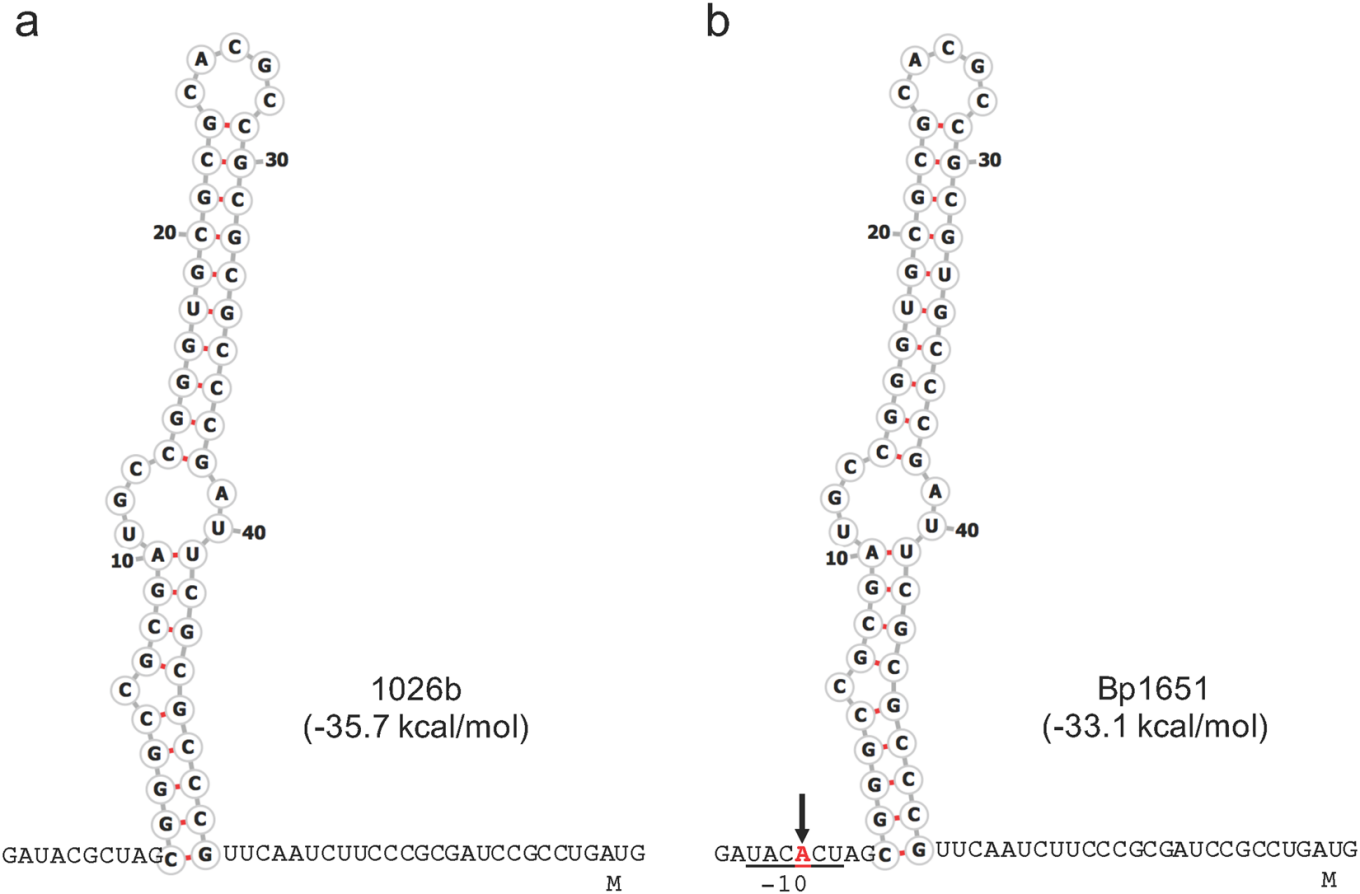
Structure of predicted Rho-independent transcriptional terminator located in the untranslated *penA* upstream region. The predicted 49 nucleotide terminator sequence (TERM 264) is situated between nucleotides 25 and 73 upstream of the *penA* gene start codon in both 1026b (**a**) and Bp1651 (**b**). The G to A transition that generates the indicated −10 promoter sequence (underlined) at position −78 with respect to the start of *penA* is indicated in red type and marked by the arrow. The secondary structure numbering refers to the location of the respective bases in the 49 nucleotide terminator sequence. Gibbs free energy values for the secondary structures are shown in parentheses.

In a quest to understand the transcriptional organization of the *penA* region on chromosome 2 we first examined its surrounding sequences. The *penA* gene is separated from the upstream *nlpD1* gene by 174 bp (Fig. 1a). NlpD1 is annotated as an outer membrane lipoprotein and contains conserved motifs present in *E*. *coli* NlpD, one of two activators of cell wall hydrolytic amidase activity^18^. This organization is conserved in closely related members of the *B*. *pseudomallei* complex, e.g. *B*. *mallei* and *B*. *thailandensis* (Fig. S1). Bioinformatic analyses did not identify any promoter sequences in the *nlpD1-penA* intergenic region (IR) or within *nlpD1*, suggesting that the two genes may form an operon transcribed from a promoter located upstream of *nlpD1*. The sequences of the IR of diverse *B*. *pseudomallei* strains are highly similar, with few conserved single nucleotide changes and a rare insertion (Fig. 1b). Surprisingly, in addition to a potential palindrome constituting a possible transcriptional terminator downstream of *penA*, we identified a 49 nucleotide palindromic sequence capable of forming a stem-loop structure in the *penA* 5′-untranslated region (5′-UTR) in the *nlpD1-penA* IR. In the *Burkholderia* Genome Database (www.burkholderia.com) 1026b entry this sequence was recently annotated as Rho-independent transcription terminator 264 (TERM 264) (Fig. 2). The average free energy reported for *E*. *coli* terminators is −10 kcal/mol^19^. By comparison, the calculated free energy for TERM 264 is high (∆G = −35.7 kcal/mol), which is comparable to that of the strong *E*. *coli* rRNA operon *rrnB* T1 Rho-independent terminator (−32.2 kcal/mol; calculated with the same software used in the present study)^20^. These observations laid the basis for comparative studies with the previously identified extensively β-lactam resistant strain Bp1651 and the CAZ^s^ strain 1026b that explore regulatory features affecting *penA* expression and contribution to β-lactam resistance.

### The *nlpD1* and *penA* genes form an operon

**Figure 3:**
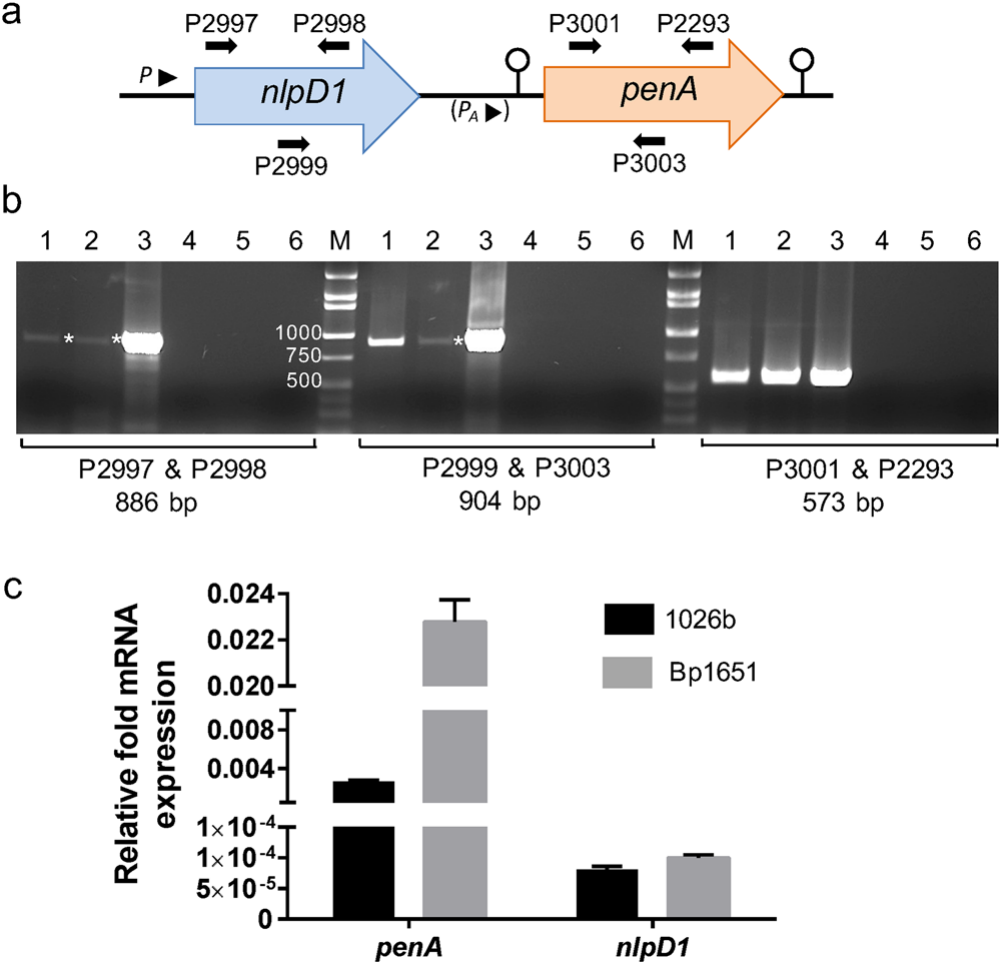
Transcriptional analysis of *nlpD1* and *penA* expression. (**a**) Map of the *nlpD1-penA* region. Primers and their relative locations are indicated with P numbers and horizontal arrows. *P*_*A*_ indicates the location of a predicted promoter in Bp1651 generated by a G to A nucleotide transition at position −78 of the IR. (**b**) RT-PCR analysis of *nlpD1* and *penA* expression. Total RNA was isolated from log phase cells grown in LB medium and converted to cDNA, which served as template for PCR with primer pairs indicated below the panels together with sizes of the expected PCR fragments. Lanes: 1, 1026b cDNA; 2, Bp1651 cDNA; 3, 1026b genomic DNA; 4, DNAse treated RNA from 1026b minus RT; 5, DNAse treated RNA from Bp1651 minus RT; 6; sterile water; M, DNA size ladder (Minnesota Molecular, Minneapolis, MN). Sizes of pertinent size ladder fragments are indicated adjacent to the left size ladder. Asterisks in lanes 1 and 2 of the left panel and lane 2 of the center panel mark weakly amplified DNA fragments. The inset is a minimally processed section of an image of a full length agarose gel shown in Fig. S5. (**c**) RT-qPCR analysis of *penA* and *nlpD1* expression. Cells of 1026b (black bars) and Bp1651 (gray bars) were grown to mid-log phase in LB medium and total RNA was isolated. The *penA* and *nlpD1* and mRNA levels were determined by RT-qPCR. The level of expression of *penA* and *nlpD1* is shown relative to 23S rRNA. Error bars indicate the standard deviation between three biological replicates.

To assess potential polycistronic transcription of *nlpD1* and *penA*, we performed RT-PCR with primers designed to cover different portions of the *nlpD1-penA* region (Fig. 3a). Utilizing cDNA derived from 1026b total RNA as template, primer pair 2999 & 3003 amplified a 904 bp fragment consisting of 336 bp of the 3′ portion of *nlpD1*, the 174 bp *nlpD1-penA* IR and 394 bp of the 5′ portion of *penA* (Fig. 3b, middle panel, lane 1). The presence of this fragment indicated that the two genes are co-transcribed, located on the same polycistronic mRNA and thus likely belong to the same operon. Fragment intensity is significantly less with cDNA derived from Bp1651 total RNA as template (Fig. 3b, middle panel, lane 2), likely because the main *penA* transcript in this strain originates from a promoter, hereafter referred to as *P*_*A*_, that arises from a G to A transition at position −78 in the IR (Figs 1b and 2b), so the cDNA derived from this transcript does not contain the P2999-binding site. The intensity of the RT band from the Bp1651 cDNA is similar to that observed with *nlpD1* cDNA (Fig. 3b, left panel) indicating that the transcripts are derived from the same promoter upstream of *nlpD1* and not very abundant. The 1026b *nlpD1-penA* IR fragment cDNA intensity is significantly higher than that observed from the 1026b cDNA *nlpD1* fragment (compare Fig. 3b, left and middle panel, lanes 1). The *penA* cDNA band intensities obtained with both 1026b and Bp1651 RNA-derived templates (Fig. 3b, right panel, lanes 1 and 2) are significantly higher when compared to those observed with *nlpD1* and *nlpD1-penA* (Fig. 3b, left and middle panel). cDNA levels of *penA* obtained with RNA from Bp1651 (Fig. 3b, right panel lane 2) were higher than those observed in 1026b (Fig. 3b, right panel, lane 1).

RT-qPCR analyses confirmed that *penA* transcript levels are >8-fold higher in Bp1651 when compared to 1026b (Fig. 3c), which is consistent with increased *penA* expression due to the presence of the *P*_*A*_ promoter. The data also confirm that *nlpD1* transcript levels are significantly lower than *penA* transcript levels in both strains.

### A locally conserved single mutation generates a *penA* promoter

Up-regulated *penA* expression was shown as a cause of CAZ^r^ over 25 years ago^9^. Several studies subsequently demonstrated the presence of a conserved G to A transition at position −78 upstream of the *penA* start codon (Figs 1b and 2b) and it was suggested that the mutation likely generates a functional −10 region that is absent in wild-type strains^10,12^. However, until now little experimental evidence supported these notions.

**Figure 4:**
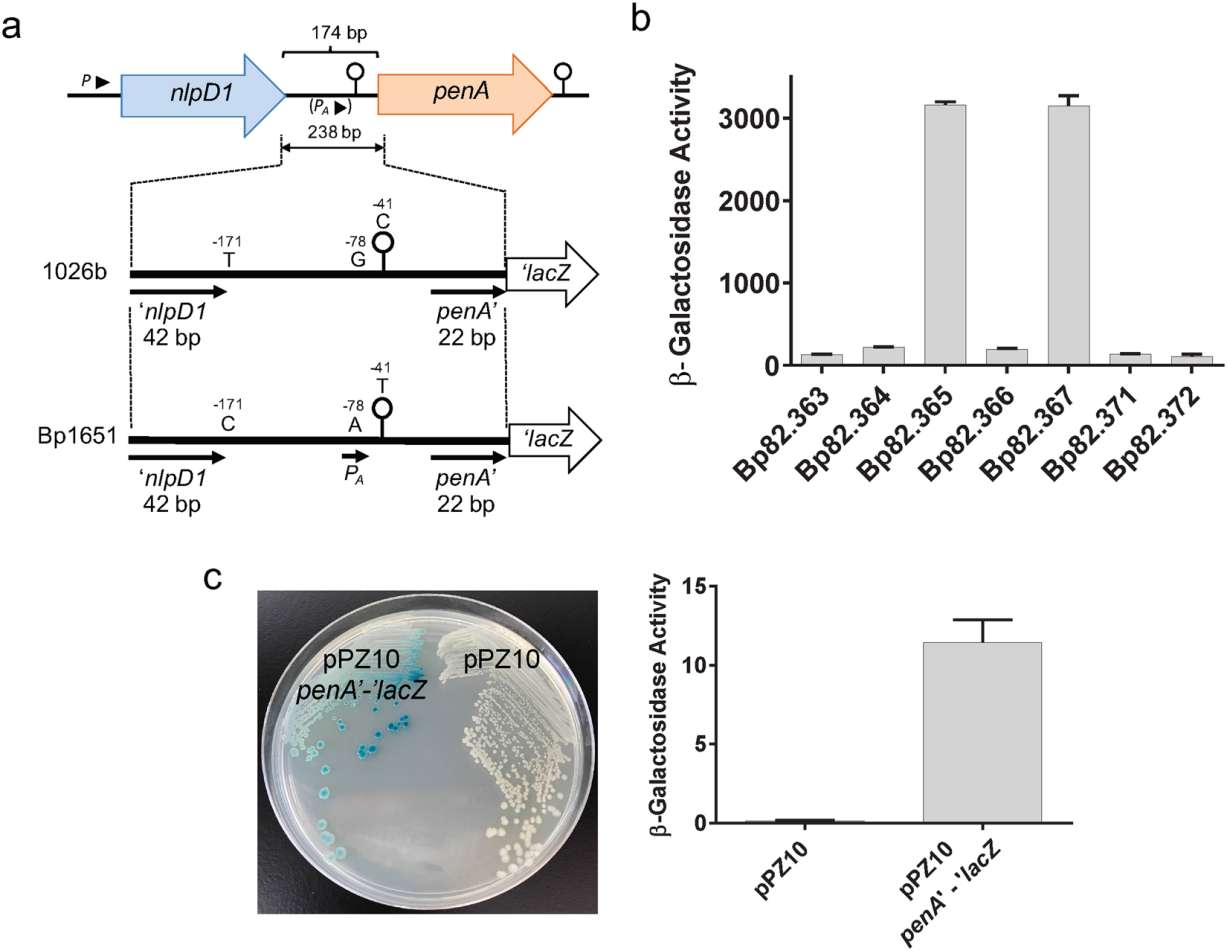
Promoter activity in native and genetically engineered *nlpD1*-*penA* intergenic regions. (**a**) Design of reporter *lacZ* reporter gene constructs. The indicated 238 bp DNA fragments shown by the thick lines (plus flanking SpeI and HindIII restriction sites) were obtained from PCR fragments amplified from 1026b and Bp1651 genomic DNA. The indicated −41C, −78G and −171T nucleotides present on the 1026b fragment were individually changed to −41T, −78A and −171C present in Bp1651. Lastly, a 532 bp DNA fragment (plus flanking SpeI and HindIII restriction sites) containing a 336 bp instead of a 42 bp *nlpD1* terminus was obtained from a PCR fragment amplified from 1026b genomic DNA. These fragments were cloned into a mini-Tn*7*-*lacZ* transcriptional fusion vector and integrated into the Bp82.27 chromosome. (**b**) β-Gal activity in strains harboring the single-copy *lacZ* reporter constructs. The strains resulting from chromosomal integration were Bp82.363 (empty vector), Bp82.365 (1026b fragment), Bp82.365 (Bp1651 fragment), Bp82.366 (1026b fragment with −41T), Bp82.367 (1026b fragment with −78A), Bp82.371 (1026b fragment with −171C) and Bp82.372 (1026b fragment with a 331 bp instead of 42 bp *nlpD1* 3′ terminus. β-Gal activities were measured in triplicate on three separate days and are expressed in Miller units. Error bars indicate standard deviation from the mean. (**c**) The *P*_*A*_ promoter is active in *E*. *coli*. A plasmid was constructed that contains a *penA*’-‘*lacZ* translational fusion under transcriptional and translation control of the 174 bp 1026b IR with the -78 G to A transition. The resulting pPZ10 *penA*’-‘*lacZ* thus contains the *P*_*A*_ promoter. The first 7 amino acids of PenA are fused in-frame to LacZ. The resulting pPZ10 *penA*’-‘*lacZ* and the empty pPZ10 vector were transformed into strain DH5α. β-Gal activity was assessed by plating on an LB plate with X-Gal indicator (left) or by measuring hydrolysis of ortho-nitrophenyl-β-D-galactopyranoside (ONPG) in culture grown cells (right). The ONPG β-Gal assays were performed in triplicate and activity is expressed in Miller units. Error bars indicate standard deviation from the mean. The plate picture is a minimally processed section of an image of an agar plate shown in Fig. S6.

The *penA* upstream regions of the CAZ susceptible (CAZ^s^) strain 1026b and the CAZ^r^ strain Bp1651 differ by point mutations at positions −171, −78, and −41 upstream of the *penA* initiation codon (Fig. 4a). To assess the possible contributions of these point mutations to *penA* expression they were individually introduced into the 1026b upstream sequence. Transcriptional *penA*’-*lacZ* fusions were then generated by cloning fragments containing the 1026b, the Bp1651 IR and the 1026b IR with the engineered Bp1651 −171C, −78A and −41T mutations into a mini-Tn*7*-*lacZ* fusion vector. The mini-Tn*7-penA*’*-lacZ* fusion containing elements were transposed into the Bp82.27 genome, along with the empty vector and β-Galactosidase (β-Gal) activity was measured (Fig. 4b). Low levels of β-Gal activity were expressed in strains harboring the empty vector (Bp82.363) or *penA*’-*lacZ* reporter constructs with the 1026b (Bp82.364 and Bp82.372; short and long, respectively), −41T (Bp82.366) and −171C (Bp.82.371) containing *penA* upstream regions. In contrast, high levels of β-Gal activity were expressed in strains harboring *penA*’-*lacZ* reporter constructs with the Bp1651 (Bp82.365) and 1026b with −78A (Bp82.367) containing *penA* upstream regions, indicating that the −78A mutation generates a promoter (*P*_*A*_) that leads to constitutive overexpression of *penA*.

*P*_*A*_ is active in *E*. *coli* since strain DH5α carrying a plasmid with a *penA*’*-lacZ* translational fusion containing the 174 bp 1026b IR with the −78A mutation and the first seven amino acids of PenA fused in-frame to LacZ expressed β-Gal activity (Fig. 4c).

### The transcriptional terminator located upstream of *penA* is functional

**Figure 5:**
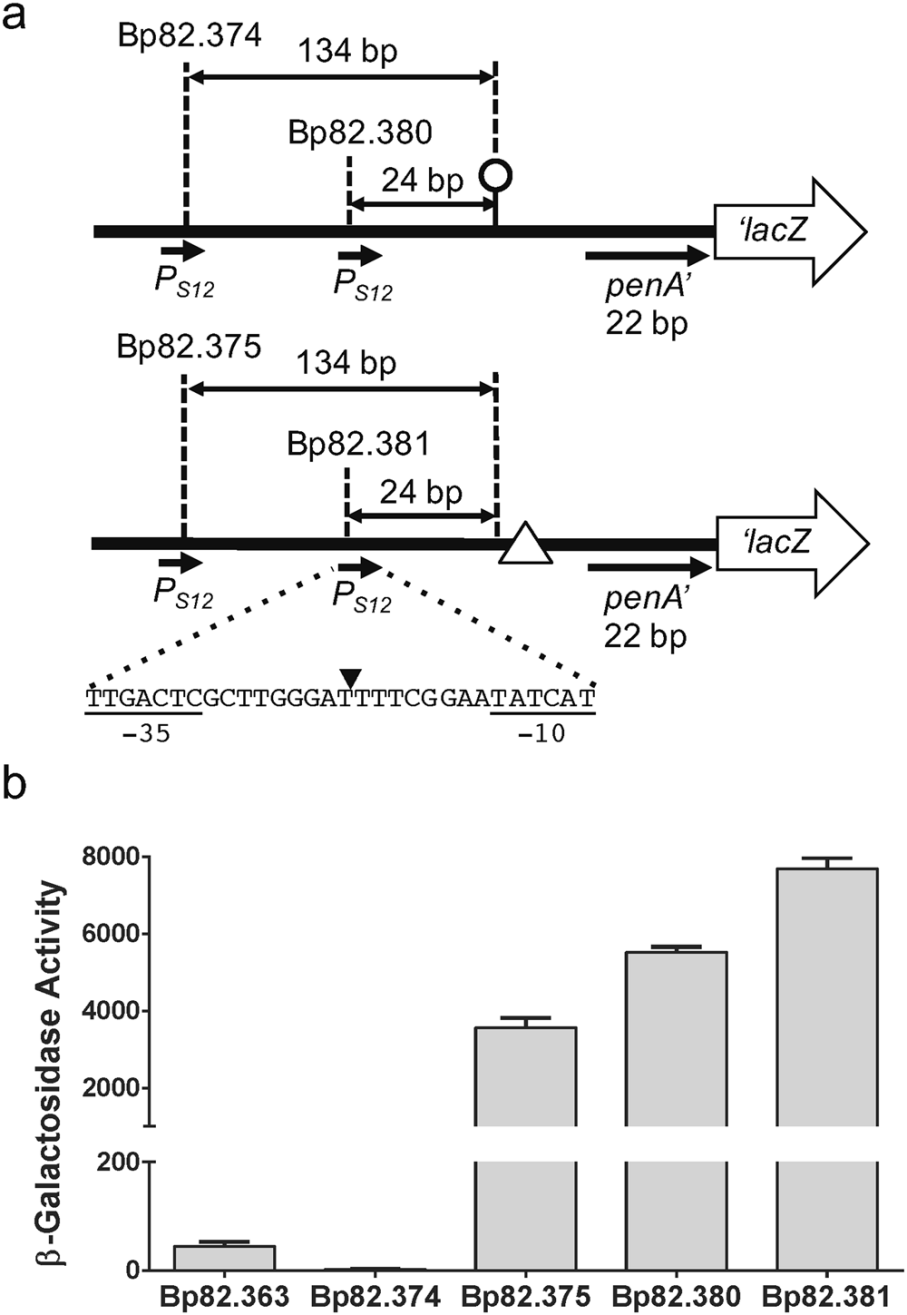
Functional analysis of the IR Rho-independent transcription terminator. (**a**) Design of reporter constructs. To assess the functionality of the putative IR transcriptional terminator TERM 264, the constitutive *B*. *thailandensis* ribosomal gene 12 promoter (*P*_*S12*_) was inserted upstream of the *penA*’*-lacZ* transcriptional fusion such that *P*_*S12*_ was centered either 134 bp or 24 bp upstream of TERM 264 (indicated by the lollipop) or 134 bp or 24 bp upstream of a clean TERM 264 deletion (indicated by Δ). The center of *P*_*S12*_ promoter sequence is marked with a filled triangle in the shown sequence. These fragments were cloned into a mini-Tn*7*-*lacZ* transcriptional fusion vector and integrated into the Bp82.27 chromosome. (**b**) β-Gal activity in strains harboring single-copy *lacZ* reporter constructs. The strains resulting from chromosomal integration of *P*_*S12*_-*penA*’-*lacZ* fusions were Bp82.374 (*P*_*S12*_ 134 bp upstream of TERM 264), Bp82.380 (*P*_*S12*_ 24 bp upstream of TERM 264), Bp82.375 (*P*_*S12*_ 134 bp upstream of ΔTERM 264) and Bp82.381 (*P*_*S12*_ 24 bp upstream of ΔTERM 264). Bp82.363 served as empty vector control. β-Gal activities were measured in triplicate on three separate days and are expressed in Miller units. Error bars indicate standard deviation from the mean.

To assess whether the predicted Rho-independent TERM 264 in the *penA* 5′-UTR functions as transcriptional terminator, constructs were engineered where the *penA*’*-lacZ* fusion is expressed from the constitutive *B*. *thailandensis* S12 promoter (*P*_*S12*_) with and without TERM 264 (Fig. 5a). The mini-Tn*7-penA*’*-lacZ* fusion containing elements were transposed into the Bp82.27 genome, along with the empty mini-Tn*7*-*lacZ* vector and β-Gal activity was measured (Fig. 5b). Placement of *P*_*S12*_ 134 bp upstream of TERM 264 (Bp82.374) completely abolished expression of β-Gal activity to levels that are below those observed with the empty vector control (Bp82.363). In contrast, placement in the same position in a construct with a clean TERM 264 deletion (Bp82.375) resulted in high levels of β-Gal activity. Bp82.380 containing *P*_*S12*_ 24 bp upstream of TER M264 expressed high levels of β-Gal activity and this activity is slightly increased in Bp82.381 where *P*_*S12*_ is placed in the same position in a construct lacking TERM 264.

### β-Lactam resistance in Bp1651 extends beyond PenA

To ascertain that *penA* is the major player in β-lactam resistance in strain Bp1651 we constructed a Δ*penA* mutant (Bp881) where the gene is deleted from its start to stop codon. When compared to Bp1651, Bp881 was significantly less resistant to CAZ (256-fold) and IPM (64-fold), and also exhibited a significantly decreased MEM MIC (4-fold) (Table 1). The results confirm that in Bp1651 PenA alone is a major player in CAZ and IPM resistance, as well as decreased MEM susceptibility.

**Table 1:** Ceftazidime and carbapenem susceptibilities of clinical *B*. *pseudomallei* isolates and mutant derivatives.

Strain	Relevant Genotype	MIC (μg/mL)^a^		
		Ceftazidime	Meropenem	Imipenem
1026b	Prototype	4	1	1
Bp1651	Parental strain^b^	256	4	16
Bp881	Δ*penA*	1	1	0.25
Bp875	*TR*7*0_2*7*6*7*-flgN* IR::T24^c^	8	0.25	1
Bp876	*dacC*::T24	8	0.25	1
Bp877	*shc*::T24	64	2	4
Bp878	*penA*::T24	2	1	0.25
Bp879	*TR*7*0_1911*::T24	4	0.25	1
Bp880	*TR*7*0_0856*::T24	128	2	2

^a^Minimal inhibitory concentration (MIC) was determined using the broth microdilution method performed in triplicate and on three separate occasions. CLSI defined cut-offs are (in μg/ml): ceftazidime ≤8 susceptible, 16 intermediate, ≥16 resistant; imipenem: ≤4 susceptible, 8 intermediate, ≥16 resistant. There are no defined cut-off values for meropenem in the CLSI guidelines for *B*. *pseudomallei*. Assuming values for other non-*Enterobacteriaceae* meropenem cut-offs can be assigned as (in μg/ml) ≤4 susceptible, 8 intermediate, ≥16 resistant.

^b^Bp1651 is the parental strain to Bp875 to Bp881.

^c^See Fig. 6 for a graphical representation of transposon insertion sites and gene function explanations.

**Figure 6:**
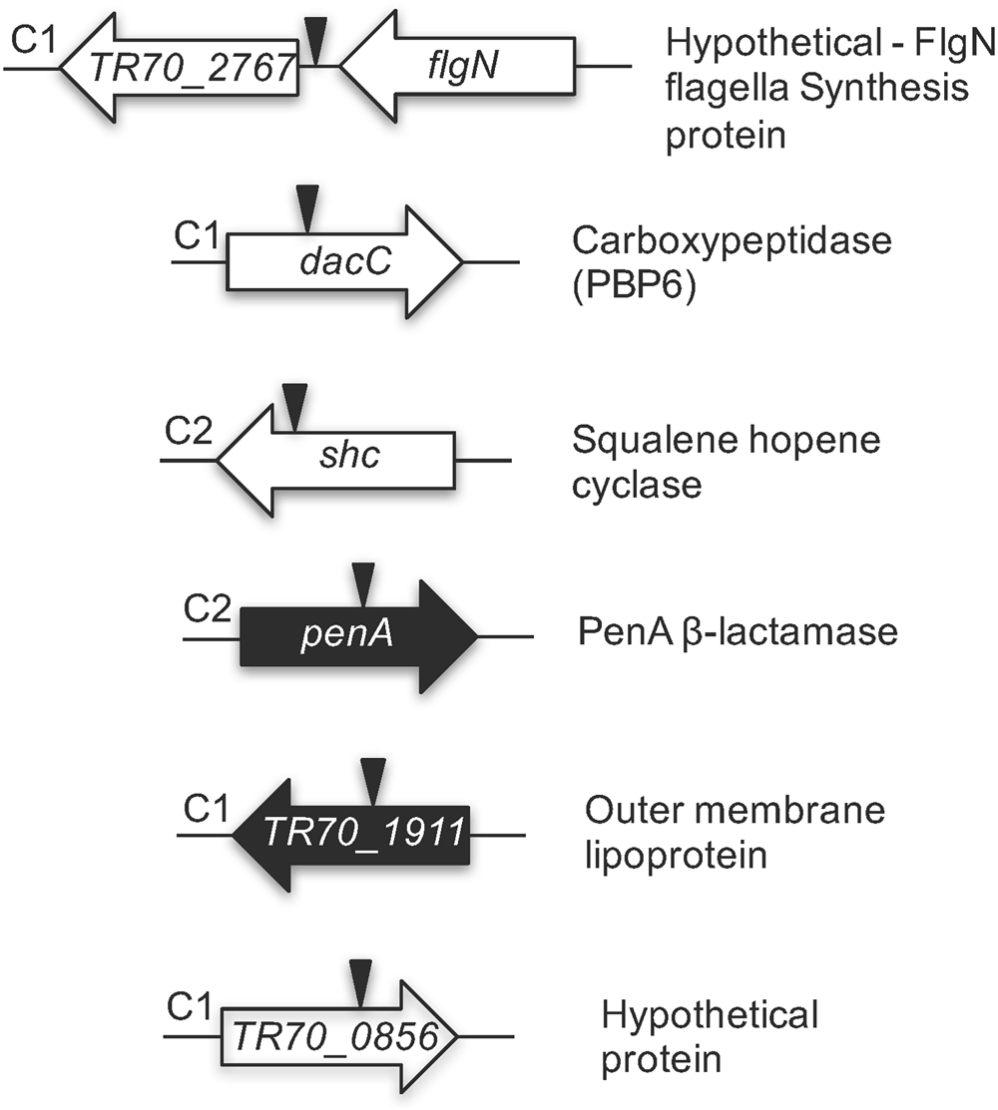
Transposon T24 insertions in Bp1651 with decreased ceftazidime and meropenem resistance. Transposon insertions were mapped to the Bp1651 genome (GenBank: CP012041.1 and CP012042.1) by determining the transposon-chromosomal DNA junction sequences, followed by BLAST. The approximate T24 insertion sites in either chromosome 1 (C1) or 2 (C2) are indicated by the vertical arrowheads. Genes indicated in black were identified in mutants that did not grow on LB plates in the presence 16 μg/ml ceftazidime or 2 μg/ml meropenem. Genes indicated in white were identified in mutants that did not grow on LB plates containing meropenem, but grew on ceftazidime-containing plates.

To assess whether PenA alone or additional determinants were involved in Bp1651 β-lactam resistance, random transposon mutagenesis was performed. Transposon mutants were screened for no growth on LB plates in the presence of 16 μg/ml CAZ or 2 μg/ml MEM. The screening identified two mutants that grew on neither CAZ nor MEM containing plates (Bp878 and Bp879) and four mutants that grew on CAZ, but not MEM containing plates (Bp875, Bp876, Bp877 and Bp880). The six mutants were further characterized by determining CAZ, MEM and IPM MICs (Table 1), and mapping the respective transposon insertion sites in the Bp1651 genome (Fig. 6). MIC testing revealed that all six mutants tested exhibited decreased CAZ and MEM resistance when compared to Bp1651, but to various degrees. Mutants that did not grow on LB plates containing either CAZ or MEM, contained insertions in *penA* (Bp878) or a gene annotated as *TR*7*0_1911* (Bp879). Both of these mutants exhibited similar decreased resistance to CAZ (64 to 128-fold) as the *penA* deletion or transposon mutants, but reduced resistance to carbapenems was more pronounced in the *TR*7*0_1911* mutant. In the majority of sequenced *B*. *pseudomallei* genomes, this gene is annotated as a lipoprotein. Mutants that did not grow on LB plates containing MEM, but grew on CAZ-containing plates exhibited various degrees of reduced carbapenem resistance when compared to Bp1651. The most pronounced differences, a 16-fold MIC reduction, with MEM and IPM were observed with mutants Bp875 and Bp876. Bp875 contained an insertion in an intergenic region between a gene encoding a hypothetical protein and putative *flgN*, encoding a flagellum assembly protein. Bp876 is a mutant with an insertion in *dacC* (D-ala-D-ala-carboxypetidase or PBP6), indicating that both MEM and IPM interact with this PBP6 in *B*. *pseudomallei*. Both mutants also exhibited a significantly (32-fold) reduced CAZ^r^. Insertions in Bp877 and Bp879 exhibited the least pronounced differences in their susceptibilities to CAZ and carbapenems. Bp877 contains an insertion in the gene for squalene hopene synthase, which catalyzes cyclization of squalene to hopene in the bacterial hopanoid biosynthetic pathway^21,22^. Bp879 contains an insertion in a gene encoding a hypothetical protein.

## Discussion

*B*. *pseudomallei* PenA β-lactamase possesses several unique molecular features. Unlike most β-lactamases the enzyme is a membrane-bound lipoprotein^23^ secreted via the twin arginine transport system^24^. These unique features extend to the transcriptional regulation of *penA* expression.

RT and RT-qPCR analyses showed: (1) In wild-type strains *penA* is co-transcribed with the upstream *nlpD1* gene. The *nlpD1-penA* transcript likely originates from a promoter located upstream of *nlpD1*. Strains carrying mini-Tn*7*-*penA*’-*lacZ* constructs containing either 336 bp (Bp82.372) or 42 bp (Bp82.364) of the *nlpD1* 3′ terminus both expressed low levels of β-Gal activity indicating absence of a promoter in this region. NlpD1 is expressed during human infection as the protein is reactive against melioidosis patient sera^25^; (2) although *nlpD1* and *penA* are co-transcribed from likely the same promoter, *nlpD1* transcript levels in LB grown cells are very low when compared to *penA*. A plausible explanation for this observation is that the *penA* portion for the *nlpD1-penA* transcript is more stable than the *nlpD1* portion, in part perhaps mediated by the stem-loop structure of TERM 264. A stem-loop structure in the 5′-UTR of *Serratia marcescens ampC* mRNA was shown to be involved in increased transcript stability^26^. The resulting PenA levels are sufficient for achieving in wild-type *B*. *pseudomallei* strains clinically significant intrinsic resistance to β-lactam antibiotics such as amoxicillin and carbenicillin, but not CAZ and carpapenems.

In this study we confirm for the first time in *B*. *pseudomallei* that the conserved G to A transition at position −78 upstream of the *penA* start codon that has been documented in CAZ^r^ clinical isolates^10–12^ indeed generates a constitutive promoter (*P*_*A*_). This leads to transcriptional *penA* overexpression and thus increased β-lactam resistance, including clinically significant CAZ^r^. Our data are consistent with the previous finding that in a laboratory-selected CAZ^r^ resistant *B*. *thailandensis* mutant this mutation leads to increased *penA* transcription by generating a −10 region that is closer to consensus (Fig. S1)^27^. Clinical *B*. *pseudomallei* isolates with *P*_*A*_ alone exhibit CAZ^r^, but at lower levels. In contrast, clinical isolates with high-level CAZ^r^ frequently contain *P*_*A*_ and PenA amino acid substitutions^10,12^.

We also show that the stem-loop structure in the *penA* 5′-UTR annotated as Rho-independent TERM 264 terminates transcription from a strong constitutive *B*. *thailandensis* promoter and that promoter placement in close proximity to TERM 264 interferes with terminator function. These findings support the notion that the conserved location of the G to A transition, and thus *P*_*A*_, immediately adjacent to TERM 264 in both *B*. *pseudomallei* and *B*. *thailandensis* (Fig. S1), possibly also interferes with terminator function. Repeated attempts aimed at determining the transcriptional start site of *penA* in mutants containing *P*_*A*_ failed because of the inability to overcome the secondary structure formed by TERM 264 sequences.

Overall, the scenario with *nlpD1-penA* is reminiscent of *E*. *coli* AmpC β-lactamase regulation. Inherited β-lactam resistance can either be due to promoter mutations or mutations affecting the *ampC* attenuator^28,29^. The *ampC* attenuator is the transcriptional terminator of the upstream *frd* operon and the *ampC* promoter is located in the last gene of the *frd* operon^30^. In this bacterium, AmpC is non-inducible, but is regulated via promoter and attenuator mechanisms, e.g. growth rate dependent anti-termination^28^. Although not yet tested, a similar mechanism of anti-termination could be at play in regulation of *B*. *pseudomallei penA* expression.

We presently do not understand whether *penA* expression is subject to regulation by factors other than promoter mutation and attenuation. Two mechanisms of induction of β-lactamase activity in Gram-negative bacteria have been described so far, sensing of β-lactams by a histidine kinase and induction of β-lactamase via a response regulator^31^ and sensing of cell wall damage and induction by cell wall components^32^. In Gram-negative bacteria, direct sensing of β-lactam antibiotics via sensor kinase is rare and the predominant mechanism of regulation of production of β-lactamase (e.g. AmpC) is induction in response to cell wall damage by β-lactam antibiotics^32^. Such damage results in release of *N*-acetylglucosamine-anhydro-*N*-acetylmuramic acid-oligopeptide. The GlcNAc-anhydro-MurNAc-oligopeptide is transported into the cell by AmpG. After release of GlcNAc, the cytoplasmic anhydro-MurNAc-oligopeptide complexes with the LysR-type transcriptional regulator AmpR to activate transcription of *ampC*. The inducing activity of anhydro-MurNAc-oligopeptide is modulated by AmpD, a cytosolic *N*-acetyl-anhydromuramyl-L-alanine amidase that cleaves the oligopeptide stems from the muropeptide. While the AmpR-AmpC system is present in *B*. *cenocepacia* (PenR-PenB), it is absent from *B*. *pseudomallei*^7^. Although *B*. *pseudomallei* genomes encode potential orthologs of AmpD (e.g. K96243 BPSL2993) and AmpG (e.g. 1710b BURPS1710b_0375^33^, their role in β-lactam resistance, if any, remains unknown; it may be restricted to cell wall recycling^32,34^. We previously noted that deletion of two LysR-type regulators present upstream of *nlpD1* and downstream of *penA* neither affected *penA* expression nor susceptibility to β-lactam antibiotics that are substrates of PenA^24^.

We also do not yet understand whether the transcriptional association of *penA* with *nlpD1* is coincidental or implies a yet to be discovered functional relationship. In *E*. *coli*^18^ and other Gram-negative bacteria, e.g. *Vibrio cholerae*^35^, NlpD and EnvC are two non-redundant activators of periplasmic amidases (three in *E*. *coli*, AmiA, AmiB and AmiC, and one in *V*. *cholerae*, AmiC) that are involved in cleavage of septal peptidoglycan, a step required for daughter cell separation in the final stages of cell division. Whereas no EnvC has been annotated in *B*. *pseudomallei*, the bacterium’s genome contains two *nlpD* genes, *nlpD1* located upstream of *penA* and *nlpD2*, located in the same genomic context as in *E*. *coli*, i.e. upstream of *rpoS* (Fig. S2). NlpD1 and NlpD2 contains the three motifs/domains also found in the *E*. *coli* NlpD homolog: (1) a lipoprotein signal sequence for outer membrane localization; (2) the lysine motif (LysM) that is common in cell envelope-associated proteins and involved in peptidoglycan-binding activity; and (3) the degenerate LytM (dLytM) domain that is required for the protein’s cell wall hydrolytic amidase activating activity^18^ (Fig. S3). Although related to metalloproteases, the carboxy-terminal dLytM domains of NlpD1 and NlpD2, like *E*. *coli* EnvC and NlpD, are missing the residues required for coordination of the catalytic Zn^2+^ ion (Fig. S4)^36^. Purified NlpD1 exhibited no protease activity^37^. Although these observations show that NlpD1 is likely a functional amidase activator with properties found in *E*. *coli* NlpD, a role in β-lactamase regulation is not readily evident. Curiously, the amidase it regulates, likely putative AmiC (e.g. BURPS1710b_1064 in 1710b or BPSL0859 in K96243^33^), is functionally equivalent to AmpD. Although localized in two separate cellular compartments, both AmpD and AmiC release peptide side chains from the glycan strand by hydrolyzing the amide bond between the *N*-terminal-L-alanine of the oligopeptide chain and MurNAc^34^.

Although our data clearly show that β-lactam resistance in Bp1651 is largely due to transcriptional overexpression of PenA at the transcriptional and post-transciptional levels, coupled with PenA structural mutations, this resistance is multifactorial and extends beyond PenA. In this strain, a low-molecular weight penicillin binding protein (PBP6) seems to play a significant role in carbapenem resistance, and to a lesser degree CAZ^r^, perhaps by affecting the balance of muropeptides in the cell wall metabolite recycling pool that has previously been shown to affect β-lactamase expression levels in several Gram-negative bacteria^34^. Inactivation of *TR*7*0_1911*, a gene encoding a lipoprotein also had a profound effect on carbapenem and CAZ^r^, perhaps because interruption of this gene compromises cell envelope integrity. In Bp1651, the gene is annotated as LptE lipopolysaccharide assembly lipoprotein. However, this annotation is likely incorrect because LptE is an essential protein, but we readily obtained a knockout mutant. Interference with the bacterial hopanoid biosynthetic pathway had a minor yet noticeable effect on CAZ and carbapenem resistance. Hopanoids are bacterial membrane triterpenoid lipids that are involved in modulation of membrane stability and impermeability of the bacterial membrane. In *B*. *cenocepacia*^38^, *B*. *pseudomallei*^39^ and *B*. *thailandensis*^40^ studies with mutants affecting various enzymatic steps in hopanoid biosynthesis and export show that these lipids confer resistance to multiple stresses, including detergent and antibiotic exposure. Intriguingly, an insertion in an intergenic region between a gene encoding a hypothetical protein and *flgN*, the gene for a flagellum assembly protein, profoundly affected carbapenem resistance and to a lesser degree CAZ^r^. A recent report indicated that decreased MEM susceptibility in clinical isolates is frequently associated with regulatory mutations that lead to increased expression of efflux pumps of the resistance nodulation cell division (RND) family^41^. Most often affected is *amrR*, the gene coding for the TetR family repressor of the operon encoding the AmrAB-OprA pump. We did not see such mutations in our transposon mutant screen because this efflux pump is not functional in Bp1651 due to a mutation affecting AmrB^12^.

Although the overall transcriptional organization of the *penA* locus and its regulatory elements are conserved among members of the *B*. *pseudomallei* group, the divergence observed with the commonly used *B*. *pseudomallei* surrogate *B*. *thailandensis* E264 is obvious but its significance, if any, remains to be assessed.

## Materials and Methods

### Bacterial strains and growth

*Escherichia coli* strains used in this study were DH5α (ThermoFisher Scientific, Waltham, MA) and XL1 Blue (Agilent Technologies, Santa Clara, CA) for routine cloning and RHO3 for conjugation^42^. *B*. *pseudomallei* strains used in this study were Bp82^43^ and its ∆(*amrAB-oprA*) derivative Bp82.27^8^, and the virulent strains 1026b^11^ and Bp1651^12^. Bp82 is an attenuated derivative of 1026b^11^ and excluded from Select Agent regulations (www.selectagents.gov/SelectAgentsandToxinsExclusions.html). Strains derived from Bp1651 and Bp82.27 are listed in Tables 1 and 2, respectively. All experiments with strain Bp82 and its derivatives were conducted at biosafety level 2 (BSL-2) with Institutional Biosafety Committee approval. Virulent *B*. *pseudomallei* strains were manipulated in Select Agent-approved BSL-3 facilities in the Rocky Mountain Regional Biosafety Laboratory at Colorado State University or at the University of Florida, using approved Select Agent-compliant procedures and protocols. Bacteria were routinely cultured in Lennox LB medium (5 g/L NaCl) at 37 °C with aeration. Bp82-derived strains were grown in LB broth or on LB agar plates supplemented with 40 or 80 μg/ml adenine, respectively. Antibiotics were used at the following concentrations: 100 μg/ml ampicillin (Ap) and 35 μg/ml kanamycin (Km) for *E*. *coli*; 350 μg/ml Km for *B*. *pseudomallei* wild-type strains and 15 μg/ml gentamicin for Δ(*amrAB-oprA*) strains. YT medium with 15% sucrose and 50 μg/ml 5-bromo-4-chloro-3-indoxyl-β-D-glucuronide (X-Gluc) (Gold Biotechnology, St. Louis, MO) was used for resolution of merodiploids during allelic exchange. For β-galactosidase based blue-white screening agar plates were supplemented with 50 μg/ml 5-bromo-4-chloro-3-indoxyl-β-D-galactopyranoside (X-Gal) (Gold Biotechnology).

**Table 2:** Bp82 and *derivatives* used in this study.

Strain	Relevant properties	Source
Bp82	Attenuated Δ*purM* derivative of 1026b	^43^
Bp82.87	Δ(*amrAB-oprA*)	^8^
**Strains used to assess promoter activity in** ***penA*** **’** ***-lacZ*** **fusions** ^**a,b**^		
Bp82.363	Bp82.87::mini-Tn*7*T-Gm-*lacZ*	This study
Bp82.364	Bp82.87::mini-Tn*7*T-Gm-1026b-42 bp *nlpD1*-IR-*penA*’-*lacZ*^c^	This study
Bp82.365	Bp82.87::mini-Tn*7*T-Gm-Bp1651-42 bp *nlpD1*-IR-*penA*’-*lacZ*	This study
Bp82.366	Bp82.87::mini-Tn*7*T-Gm-1026b-42 bp *nlpD1*-41T-IR-*penA*’-*lacZ*	This study
Bp82.367	Bp82.87::mini-Tn*7*T-Gm-1026b-42 bp *nlpD1*-78A-IR-*penA*’-*lacZ*	This study
Bp82.371	Bp82.87::mini-Tn*7*T-Gm-1026b-42 bp *nlpD1*-171C-IR-*penA*’-*lacZ*	This study
Bp82.372	Bp82.87::mini-Tn*7*T-Gm-1026b-336 bp *nlpD1*-IR-*penA*’-*lacZ*	This study
**Strains used to assess TERM 264 terminator activity** ^**b,d**^		
Bp82.374	Bp82.87::mini-Tn*7*T-Gm-134 bp-TERM 264-*penA*’-‘*lacZ*;	This study
Bp82.375	Bp82.87::mini-Tn*7*T-Gm-134 bp-ΔTERM 264-*penA*’-‘*lacZ*	This study
Bp82.380	Bp82.87::mini-Tn*7*T-Gm-24 bp-TERM 264-*penA*’-‘*lacZ*	This study
Bp82.381	Bp82.87::mini-Tn*7*T-Gm-24 bp-ΔTERM 264-*penA*’-‘*lacZ*	This study

^a^The strains contained the mini-Tn*7*-*penA*’*-lacZ* fusion construct at *att*Tn*7 glmS*3, except Bp82.371 and Bp82.372, which had the fusion constructs inserted at *att*Tn*7 glmS*2.

^b^Gm, gentamicin resistance marker.

^c^IR, *nlpD1-penA* intergenic region.

^d^All strains contained the mini-Tn*7*-*penA*’*-lacZ* fusion construct at *att*Tn*7 glmS*3.

### MIC determination

*B*. *pseudomallei* strains were grown in cation-adjusted Mueller-Hinton II broth (MHB) (Becton Dickinson and Company, Sparks, MD) supplemented with 40 μg/ml adenine for Bp82 derivatives. The MICs of antibiotics were determined by the standard microdilution method, following CLSI guidelines^44^.

### Construction of strains with chromosomally integrated *penA*’*-lacZ* transcriptional fusions

A 262 bp fragment encompassing 42 bp of the 3′ terminus of *nlpD1*, the 174 bp *nlpD1-penA* intergenic region (IR), and 22 bp of the 5′ terminus of *penA* was PCR amplified from genomic DNA of 1026b and Bp1651 using P2978 & P2994 (primers were purchased from Integrated DNA Technologies, Coralville, IA and are listed in Table S1), and Q5 High-Fidelity DNA Polymerase according to the manufacturer’s recommendations (New England Biolabs, Ipswich, MA.). Similarly, a 556 bp PCR fragment containing an extended 336 bp *nlpD1* 3′ terminus, the 174 bp *nlpD1-penA* IR, and 22 bp of the 5′ terminus of *penA* was PCR amplified from genomic DNA of 1026b using P3005 & P3004 and Q5 High-Fidelity DNA Polymerase.

For construction of delivery vectors containing transcriptional *lacZ* fusions on mini-Tn*7* elements, the PCR fragments were digested with SpeI + HindIII and 250 bp fragments ligated between the same sites of pUC18-mini-Tn*7*T-Gm-*lacZ*^45^ to yield pPS3299 (1026b insert) and pPS3300 (Bp1651 insert). Similarly, a 544 bp SpeI-HindIII fragment containing an extended *nlpD1* 3′ terminus was cloned into pUC18-mini-Tn*7*T-Gm-*lacZ* to form pPS3319. The 262 bp PCR fragment derived from the 1026b template was also cloned into pGEM-T Easy (Promega, Madison, WI) that was A tailed with Taq polymerase (New England Biolabs) to obtain pPS3295. This plasmid was used as the template for introduction of single nucleotide changes present in the IR of Bp1651 but not 1026b (i.e. −41T, −78A and −171C; coordinates are relative to the first *penA* nucleotide) using mutagenic primers and the QuikChange II kit (Agilent Technologies). Using this strategy, pPS3296 containing −78A in the IR was created with mutagenic primer pair P2990 & P2991, pPS3297 containing −41T with P2992 & P2993, and pPS3313 containing −171C with P3006 & P3007. To derive delivery vectors containing transcriptional *lacZ* fusions on mini-Tn*7* elements, 250 bp SpeI-HindIII fragments were isolated from these plasmids and ligated between the same sites of pUC18-mini-Tn*7*T-Gm-*lacZ* to yield pPS3301 (1026b IR with −41T mutation), pPS3302 (1026b IR with −78G mutation), and pPS3318 (1026b IR with −171C mutation).

Mini-Tn*7*-*lacZ* constructs were transferred into the Bp82.27 genome by co-electroporation of the delivery vectors with the helper plasmid pTNS3, and insertions were verified by PCR and sequencing using previously described methods^46^. The strains thus obtained were: Bp82.363 with empty mini-Tn*7*T-Gm-*lacZ*; Bp82.364 with mini-Tn*7*T-Gm-1026b-42 bp *nlpD1*-IR-*penA*’-*lacZ*; Bp82.365 with mini-Tn*7*T-Gm-Bp1651-42 bp *nlpD1*-IR-*penA*’-*lacZ*; Bp82.366 with mini-Tn*7*T-Gm-1026b-42 bp *nlpD1*-41T-IR-*penA*’-*lacZ*; Bp82.367 with mini-Tn*7*T-Gm-1026b-42 bp *nlpD1*-78A-IR-*penA*’-*lacZ*; Bp82.371 with mini-Tn*7*T-Gm-1026b-42 bp *nlpD1*-171C-IR-*penA*’-*lacZ*; and Bp82.372 with mini-Tn*7*T-Gm-1026b-336 bp *nlpD1*-IR-*penA*’-*lacZ*. The strains contained the mini-Tn*7*-*penA*’*-lacZ* fusion construct at *att*Tn*7* *glmS*3, except Bp82.371 and Bp82.372, which had the fusion constructs inserted at *att*Tn*7* *glmS*2.

For terminator activity assays, gBlocks^®^ Gene Fragments were designed and purchased from Integrated DNA Technologies. These fragments contained the constitutive *B*. *thailandensis* S12 promoter (*P*_*S12*_)^46^, 93 bp (with terminator TERM264) or 44 bp (without TERM 264; ΔTERM 264) *penA* upstream region, the first 22 bp of *penA*, and 92 bp of inert “spacer DNA”. The first set of gBlocks contained *P*_*S12*_ centered 134 bp upstream of TERM 264 or its deletion junction. The respective 274 bp and 225 bp double-stranded gBlocks were cloned into A tailed pGEM-T Easy to obtain pPS3322 and pPS3323, respectively. The second set of gBlocks were of the same size and sequence as the first set but contained *P*_*S12*_ centered 24 bp upstream of TERM264 or its deletion junction. The respective gBlocks were cloned into A tailed pGEM-T Easy to obtain pPS3344 and pPS3349, respectively. To derive delivery vectors containing transcriptional *lacZ* fusions on mini-Tn*7* elements, 262 bp (with TERM 264) or 213 bp (ΔTERM 264) SpeI-HindIII fragments were isolated from these plasmids and ligated between the same sites of pUC18-mini-Tn*7*T-Gm-*lacZ* to yield pPS3331 (*P*_*S12*_ 134 bp upstream of TERM 264), pPS3332 (*P*_*S12*_ 134 bp upstream of ΔTERM 264), pPS3345 (*P*_*S12*_ 24 bp upstream of TERM 264), and pPS3350 (*P*_*S12*_ 24 bp upstream of ΔTERM 264). Mini-Tn*7*-*lacZ* constructs were transferred into the Bp82.27 genome, and insertions were verified by PCR and sequencing. The strains thus obtained were: Bp82.363 with empty mini-Tn*7*T-Gm-*lacZ*; Bp82.374 with mini-Tn*7*T-Gm-134 bp-TERM 264-*penA*’-*lacZ*; Bp82.375 with mini-Tn*7*T-Gm-134 bp-ΔTERM 264-*penA*’-*lacZ*; Bp82.380 with mini-Tn*7*T-Gm-24 bp-TERM 264-*penA*’-*lacZ*; and Bp82.381 with mini-Tn*7*T-Gm-24 bp-ΔTERM264-*penA*’-‘*lacZ*. All strains contained the mini-Tn*7*-*penA*’*-lacZ* fusion construct at *att*Tn*7* *glmS*3.

#### Construction of a translational *penA*’-‘*lacZ* fusion

To assess functionality of *penA* transcription and translation initiation signals in *E*. *coli*, a *penA*’-‘*lacZ* translational fusion was constructed. This was achieved by cloning a 292 bp HindIII-SalI fragment from pPS3296 containing the 174 bp 1026b IR with the −78A mutation between the same sites of the translational fusion vector pPZ10^47^. This step fused the first 7 amino acids of PenA in-frame to LacZ and placed the expression of the fusion protein under the control of the PenA transcription and translation initiation signals.

### β-Galactosidase assays

β-Galactosidase (β-Gal) activity was measured, and activity units were determined by the Miller method using LB-grown mid-log phase bacterial cultures in which cells were made permeable by SDS and chloroform treatment^48^.

### RT-PCR

Bacteria were grown at 37 °C in LB medium to the mid-log phase (OD_600nm_ = 0.6 to 0.8). Total RNA was isolated using the RNeasy Protect Bacteria mini kit (Qiagen, Valencia, CA) and cDNA synthesis was performed as previously described^49,50^. The transcript containing the 3′ end of *nlpD* and the 5′ end of *penA* was amplified using Q5 High-Fidelity Polymerase according to the manufacturer’s recommendations using primers P2999 & P3003. Copy DNAs derived from transcripts internal to *nlpD1* and *penA* were amplified with P2997 & P2998 and P3001 & P2293), respectively. Genomic DNA isolated from 1026b cells using the PureGene Core kit A (QIAGEN, Valencia, CA) was used as a positive control reaction, while DNAse treated cDNA generated from 1026b and Bp1651 RNA and sterile water were used as negative control templates.

### RT-qPCR

Cells were grown to mid-log phase and total RNA was extracted as described above. cDNA synthesis and reverse transcription-quantitative PCR (RT-qPCR) was performed as previously described^49,50^ to assess expression levels of *penA* and *nlpD1* mRNAs. 23S rRNA was used as the housekeeping control. The primer sets used were P2988 & P2989 for 23S rRNA^49^, P2853 & P2854 for *penA*, and P3008 & P3009 for *nlpD1*.

### Construction of a markerless chromosomal *penA* deletion

A construct for isolation of a markerless chromosomal *penA* deletion was assembled in several steps. Using genomic DNA of Bp1651 as a template and Q5 High-Fidelity DNA polymerase two partially overlapping PCR fragments were obtained: a 610 bp fragment using the upstream primer P2917 & SOEing primer P2918 and a 747 bp fragment using SOEing primer P2919 & the downstream primer P2920. The two partially overlapping fragments were then used as templates in a second PCR with Platinum Taq DNA polymerase and P2917 & P2920 to generate a 1,297 bp fragment containing a complete *penA* deletion including its start and stop codon, flanked by 585 bp of upstream and 712 bp of downstream sequences. This fragment was ligated into pGEM-T Easy to form pPS3401. Next, a 1,325 bp EcoRI fragment was isolated from this plasmid in ligated into the EcoRI site of pEXKm5 to yield pPS3402. This plasmid was then used to delete the 888 bp *penA* gene in Bp1651 by allelic exchange and sucrose counter-selection as described previously^42^.

### Transposon mutagenesis

Using a high-efficiency mating protocol originally developed for *P*. *aeruginosa*, we performed mutagenesis of strain Bp1651 using Tn*5-*based transposon T24^51^. Bp1651 was grown overnight at 37 °C in LB medium. The next day, an equal volume of 20 mM NaNO_3_ was added and the culture incubated at 42 °C for at least 3 hours. A concentrated aliquot of this culture was then mixed with a concentrated aliquot of log phase donor cells (in this case *E*. *coli* RHO3 with T24 delivery plasmid pLG107), followed by overnight incubation at 37 °C. Dilutions were then plated on LB + 300 μg/ml Km to select for transformants containing chromosomally integrated T24 transposons. This method yielded >10,000 colonies per 0.5 ml mating mixture. Kanamycin resistant colonies were picked and arrayed into 96-well plates containing LB + 35 μg/ml Km + 10% glycerol using a QPix2 colony-picking robot (Genetix). Plates were incubated for 36 h at 37 °C, followed by 1–2 h at room temperature, and then stored at −80 °C. For determination of ceftazidime and meropenem susceptibilities bacteria were replicated onto freshly prepared LB plates with 16 μg/ml ceftazidime or 2 μg/ml meropenem.

### Mapping of chromosomal T24 insertion sites

T24 insertion sites in ceftazidime and meropenem susceptible mutants were identified by semi-degenerate PCR and sequencing of the transposon-genome junctions as previously described for *B*. *thailandensis* with minor modifications^52^. KAPA polymerase was used for all steps and so pre-PCR was omitted. Template for PCR round 1 was instead obtained by mixing 5 μl of overnight culture with 5 μl of 1xKAPA GC buffer containing 2 mM MgCl_2_, followed by a 20 min incubation at 98 °C in a thermocycler. One μl of the cell lysate was then used as template in PCR round 1. After PCR round 2 and clean up, 2–4 μl of the clean-up product were mixed with 2.5 μl of 5 μM lacZ124L2 sequencing primer (5′-CAGTCACGACGTTGTAAAACGACG), and the volume was brought up to 20 μl with sterile water. Transposon-chromosomal junction DNA sequences were determined by Sanger sequencing. Transposon insertion sites were identified by BLAST searches against the Bp1651 genome sequence (Genbank accession numbers CP012041.1 and CP012042.1).

### Bioinformatic analysis

CLUSTAL multiple sequence alignment was performed by MUSCLE (3.8) on the European Bioinformatics Institute webserver at https://www.ebi.ac.uk/Tools/msa/muscle/. The RNA secondary structure plots were generated and free energy values calculated using the RNAfold WebServer (http://rna.tbi.univie.ac.at/cgi-bin/RNAWebSuite/RNAfold.cgi). BPROM available at www.softberry.com was used for promoter prediction. Terminator prediction was according to Kingsford *et al*.^53^.

### Data availability

The datasets generated and/or analyzed during the current study are available from the corresponding author upon reasonable request.

## Electronic supplementary material

Supplementary Materials
