# Supplementary material for: Transcriptional and post-transcriptional regulation of PenA β-lactamase in acquired Burkholderia pseudomallei β-lactam resistance
Source: Sci Rep. 2018 Jul 13;8:10652. doi: 10.1038/s41598-018-28843-7 (PMC6045580; doi:10.1038/s41598-018-28843-7)
Supplement: Supplementary file 1 — Supplementary Materials [file 41598_2018_28843_MOESM1_ESM.pdf]

Supplementary Materials

Transcriptional and post-transcriptional regulation of PenA β-lactamase in acquired *Burkholderia pseudomallei* β-lactam resistance

Sunisa Chirakul, Michael H. Norris, Nawarat Somprasong, Sirawit Pagdepanichkit, Linnell B. Randall, James F. Shirley, Bradley R. Borlee, Olga Lomovskaya, Apichai Tuanyok, Herbert P. Schweizer

Supplementary Table S1. Primers used in this study.
Supplementary Figure S1. Genomic organization of the *B. pseudomallei, B. mallei* and *B. thailandensis nlpD1* and *penA* loci and their features.
Supplementary Figure S2. Genomic organization of *B. pseudomallei nlpD1* and *nlpD2*.
Supplementary Figure S3. Domain organization of *E. coli* and *B. pseudomallei* NlpD.
Supplementary Figure S4. Alignment of LytM domains.
Supplementary Figure S5. Full size agarose gel of RT-PCR analysis of *nlpD1* and *penA* expression.
Supplementary Figure S6. Full size picture of *E. coli* strain on agar plate containing ß-galactosidase indicator.

Table S1. Primers used in this study.

Primer	Sequence[a,b,c]	Source
**β-galactosidase (*lacZ)* transcriptional fusion construction**		
2990	5’-TGACGAGAGCTGATACaCTAGCGGGCCG	This study
2991	5’-CGGCCCGCTAGtGTATCAGCTCTCGTCA	This study
2992	5’-CGCACGCCGCGtGCCCGATTCGC	This study
2993	5’-GCGAATCGGGCaCGCGGCGTGCG	This study
2978	5’-GCGAGC**AAGCTT**GGCGCAACGGAGA (*Hin*dIII)	This study
2994	5’-AAGCCC**ACTAGT**GTCAATCCGATGC (*Spe*I)	This study
3004	5’-AAGCCC**ACTAGT**GAAACGTTCCAGCCCGGC (*Spe*I)	This study
3005	5’-GCGAGC**AAGCTT**GGCGCAACGGAGAATGAT (*Hin*dIII)	This study
3006	5’-AGGGCTAGCGGcGTGCCCGCCGC	This study
3007	5’-GCGGCGGGCACgCCGCTAGCCCT	This study
**Primers for markerless chromosomal *penA* deletion**		
2917	5’-CATCGGGTCGTCCAGGAAACGAAG	This study
2918	5’-CCGAACGGCGCGCGGCCGCGCGCGCCAGGC<br>GGATCGCGGGAAGATTGAACGGGCGCGAAT	This study
2919	5’-ATTCGCGCCCGTTCAATCTTCCCGCGATCCGC<br>CTGGCGCGCGCGGCCGCGCGCCGTTCGG	This study
2920	5’-CGACGACGGGCTCGAAAAACAGG	This study
**RT-PCR primers**		
2293	5’-CGAGCAGCCGGTGCACGCT	This study
2997	5’-GAGATCGACCGCAGTGTC	This study
2998	5’-CGTCGCGCCGCACCTCGA	This study
2999	5’-GAAACGTTCCAGCCCGGC	This study
3001	5’-TGCTCGTCGCAGCCATTTCC	This study
3003	5’-TCGCCGCGCACAGCTCGG	This study
**Real time PCR primers**		
Bp23S_F	5’-GTAGACCCGAAACCAGGTGA	[1]
Bp23S_R	5’-CACCCCTATCCACAGCTCAT	[1]
2853	5’-TTCCCGTTCTGCAGCAC (*penA*)	This study
2854	5’-CGAATAGCGGATGAGATCGC (*penA*)	This study
3008	5’-GATTTTCTGACCGCTTACGC (*nlpD1*)	This study
3009	5’-GCATCGGATTGACGGGCTTG (*nlpD1*)	This study

[a]Lower case type indicates an introduced nucleotide change.
[b]Bold indicates a newly generated restriction enzyme cleavage site.
[c]Underline indicates a homologous splicing region.

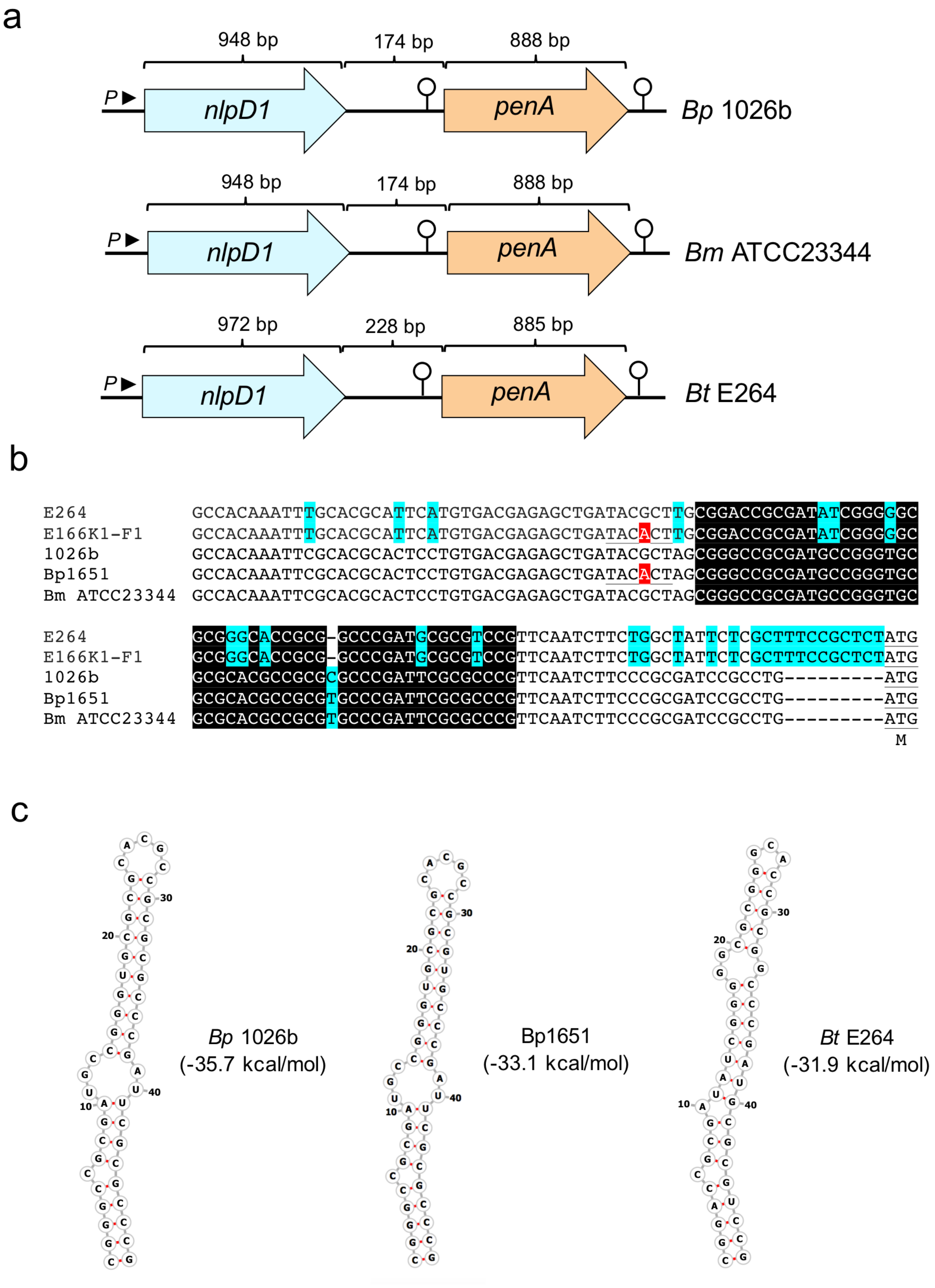
a
948 bp
174 bp
888 bp
P
nlpD1
penA
Bp 1026b
948 bp
174 bp
888 bp
P
nlpD1
penA
Bm ATCC23344
972 bp
228 bp
885 bp
P
nlpD1
penA
Bt E264
b
E264 GCCACAAATTTGCACGCATTCATGTGACGAGAGCTGATACGCTTGCGGACCGCGATATCGGGGGC
E166K1-F1 GCCACAAATTTGCACGCATTCATGTGACGAGAGCTGATACACTTGCGGACCGCGATATCGGGGGC
1026b GCCACAAATTCGCACGCACTCCTGTGACGAGAGCTGATACGCTAGCGGGCCGCGATGCCGGGTGC
Bp1651 GCCACAAATTCGCACGCACTCCTGTGACGAGAGCTGATACACTAGCGGGCCGCGATGCCGGGTGC
Bm ATCC23344 GCCACAAATTCGCACGCACTCCTGTGACGAGAGCTGATACGCTAGCGGGCCGCGATGCCGGGTGC
E264 GCGGGCACCGCG-GCCCGATGCGCGTCCGTTCAATCTTCTGGCTATTCTCGCTTTCCGCTCTATG
E166K1-F1 GCGGGCACCGCG-GCCCGATGCGCGTCCGTTCAATCTTCTGGCTATTCTCGCTTTCCGCTCTATG
1026b GCGCACGCCGCGCGCCCGATTCGCGCCCGTTCAATCTTCCCGCGATCCGCCTG---------ATG
Bp1651 GCGCACGCCGCGTGCCCGATTCGCGCCCGTTCAATCTTCCCGCGATCCGCCTG---------ATG
Bm ATCC23344 GCGCACGCCGCGTGCCCGATTCGCGCCCGTTCAATCTTCCCGCGATCCGCCTG---------ATG
M
c
Bp 1026b
(-35.7 kcal/mol)
Bp1651
(-33.1 kcal/mol)
Bt E264
(-31.9 kcal/mol)

Figure S1. Genomic organization of the *B. pseudomallei, B. mallei* and *B. thailandensis nlpD1* and *penA* loci and their features. a. In each species the *nlpD1* and *penA* genes are predicted to form an operon that is located on chromosome 2. In *B. pseudomallei* strain 1026b and *B. mallei* strain ATCC23344 the *nlpD1* and *penA* genes and their intergenic region are identical in size and over the encompassing 2,010 bp exhibit 99% sequence identity (2,002 of 2,010 bp) with no gaps. Lollipop structures indicate transcriptional terminators. Sequence data were retrieved from the *Burkholderia* Genome database (http://www.burkholderia.com)[2]. Although closely related, the *B. thailandensis nlpD1* and *penA* genes and their intergenic region differ from *B. pseudomallei* and *B. mallei* in size and sequence similarity (86% identity over 2,085 bp with numerous gaps). **b.** Main features of the *penA* 5' untranslated regions of ceftazidime susceptible (1026b) and resistant (Bp1651) *B. pseudomallei* clinical strains, ceftazidime susceptible (E264) and resistant (E166K1-F1) *B. thailandensis* strains and the ceftazidime susceptible *B. mallei* ATCC23344 type strain. The G to A mutation resulting in a constitutive *penA* promoter in ceftazidime resistant clinical *B. pseudomallei* strain Bp1651 and laboratory-selected ceftazidime resistant *B. thailandensis* strain E166K1-F1 is highlighted in red. As previously hypothesized, the G to A transition changed a putative weak 5'-TACGCT σ_{70} promoter -10 sequence that is present in CAZ susceptible strains to 5'-TACACT (underlined), a sequence that is closer to a σ_{70} promoter -10 consensus sequence (5'-TATAAT).[3,4] Nucleotides forming a stem-loop structure, i.e. a transcriptional terminator, are boxed in black. Nucleotides that differ between strains are highlighted in turquoise. **c.** Structures of the predicted terminator sequences in the *penA* 5' untranslated regions of *B. pseudomallei* (Bp) 1026b and *B. thailandensis* (Bt) E264. The structures differ because of the nucleotide sequences dffrences in this region, which includes a one-nucleotide deletion (see panel **b**). The RNA secondary structure plots were generated and free energies calculated using the RNAfold WebServer (http://rna.tbi.univie.ac.at/cgi-bin/RNAWebSuite/RNAfold.cgi). The secondary structure numbering refers to the location of the respective bases in the 49 (Bp) and 48 (Bt) nucleotide terminator sequences. The sequence (see panel **b**) and thus structure of the *B. mallei* ATCC23344 terminator is the same as that shown for *B. pseudomallei* Bp1651. Numbers in parentheses are free energy values for the respective stem-loop structures.

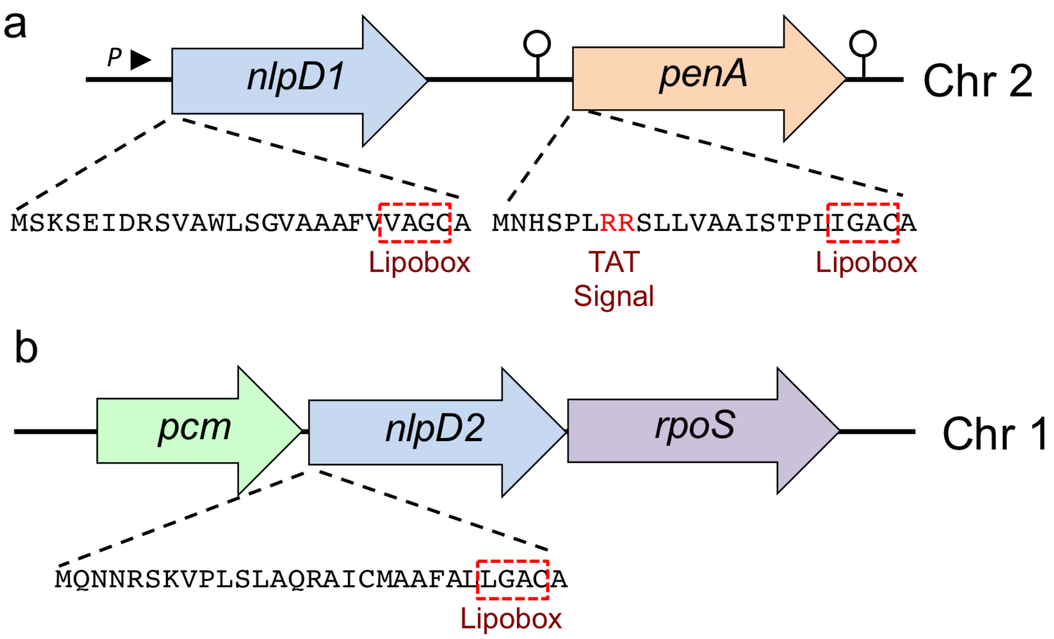

Figure S2. Genomic organization of *B. pseudomallei nlpD1* and *nlpD2*. a. The *nlpD1* and *penA* genes form an operon that is located on chromosome 2. The *penA* gene encodes a protein with twin arginine transport (TAT) signal that directs its export via the TAT system[5]. During export PenA is triacylated at the conserved lipobox cysteine[6]. The *nlpD1* gene encodes a protein with a lipobox in the amino-terminal sequences and is thus likely a lipoprotein in its mature form. NlpD1 is possibly a TAT secreted protein because in some instances a single arginine, in this case R8, is sufficient for TAT secretion[5]. **b.** The *nlpD2* gene is located on chromosome 1 and organized in the same genetic context and transcriptional organization as the *E. coli nlpD* gene. It is upstream of *rpoS* that encodes a stationary phase sigma factor and downstream of *pcm* that codes for a protein-L-aspartate O-methyl transferase. The *nlpD2* gene encodes a protein with a lipobox in the amino-terminal sequences and, similar to NlpD1, is likely a lipoprotein in its mature form. PenA, NlpD1 and NlpD2 are predicted to be located in the outer membrane because the three proteins contain alanine rather than aspartic acid in the +2 position of the signal peptidase II cleavage site, a constellation that predicts localization to the outer rather than inner membrane[7,8]. Lollipop structures indicate transcriptional terminators.

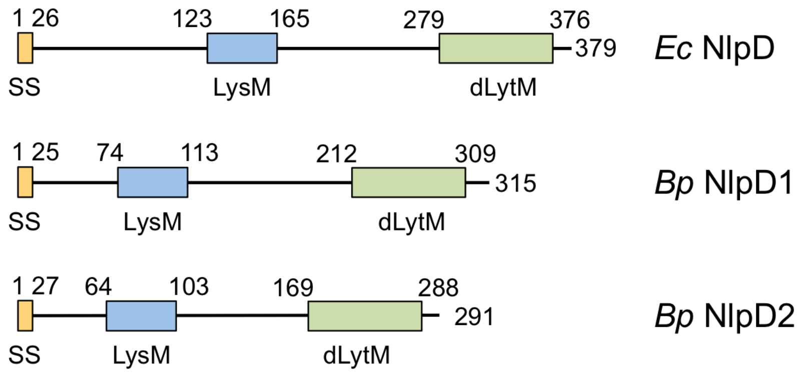

Figure S3. Domain organization of *E. coli* and *B. pseudomallei* NlpD. Indicated are lipoprotein signal sequences (SS), the lysine motif (LysM) that is common in cell envelope-associated proteins and involved in peptidoglycan-binding activity; and 3) the degenerate LytM (dLytM) domain that is required for the protein's cell wall hydrolytic amidase activating activity[9]. Domain assignments and coordinates for *E. coli* NlpD were adapted from Tsang et al.[9].

```
Bp NlpD1 198 PAKGAVVETF--QPGR---NRGIRIVGRAGDPVRAAASGRVMYAGTGLNG
Bp NlpD2 177 PVRGALLNTF--DDSK---NKGINIGGPAGEAVKAAADGRVVYAGNGLRG
Ec NlpD  263 PTEGKVIETFGASEGG---NKGIDIAGSKGQAIIATADGRVVYAGNALRG
Ec EnvC  301 PVRGPTLHRYGEQLQGELRWKGMVIGASEGTEVKAIADGRVILA-DWLQG
Sa LytM  198 LTSRKQLQPYGQYHGGG-AHYGVDYAMPENSPVYSLTDGTVVQAGWSNYG

Bp NlpD1 243 YGTLILVQHNA-DFLTAYAHNRKVLVKTGDVVQQGEQIAEMGTGDSTRAG
Bp NlpD2 222 YGNLIIIKHDA-TYLTAYAHNRALMVKEGDAVTKGQKIAEMGNSDSDRV-
Ec NlpD  310 YGNLIIIKHND-DYLSAYAHNDTMLVREQQEVKAGQKIATMGSTG-TSST
Ec EnvC  350 YGLVVVVEHGK-GDMSLYGYNQSALVSVGSQVRAGQPIALVGSSGGQGRP
Sa LytM  247 GGNQVTIKEANSNNYQWYMHNNRLTVSAGDKVKAGDQIAYSGSTGNSTAP

Bp NlpD1 292 ML-FEVRRDGK----PVNPMPYLASRQQG*
Bp NlpD2 270 MLHFEVRRQGK----PVDPLKYLPPQ*
Ec NlpD  358 RLHFEIRYKGK----SVNPLRYLPQR*
Ec EnvC  399 SLYFEIRRQGQ----AVNPQPWLGR*
Sa LytM  297 HVHFQRMSGGIGNQYAVDPTSYLQSR*
```

Figure S4. Alignment of LytM domains. The LytM domains are of *S. aureus* LytM, *E. coli* EnvC and NlpD, and *B. pseudomallei* NlpD1 and NlpD2 were aligned using CLUSTAL multiple sequence alignment by MUSCLE (3.8) on the European Bioinformatics Institute webserver at https//:www.ebi.ac.uk/Tools/msa/muscle. Residues of LytM thought to be involved in Zn^{2+} binding and catalysis are highlighted in red[10]. These residues are either altered or missing from EnvC and NlpD. Black and grey highlights indicate identical or similar amino residues, respectively. Asterisk mark stop codons following the carboxy-terminal amino acids. Sequences for *E. coli* NlpD, EnvZ and *S. aureus* LytM are adapted from Uehara et al.[10].

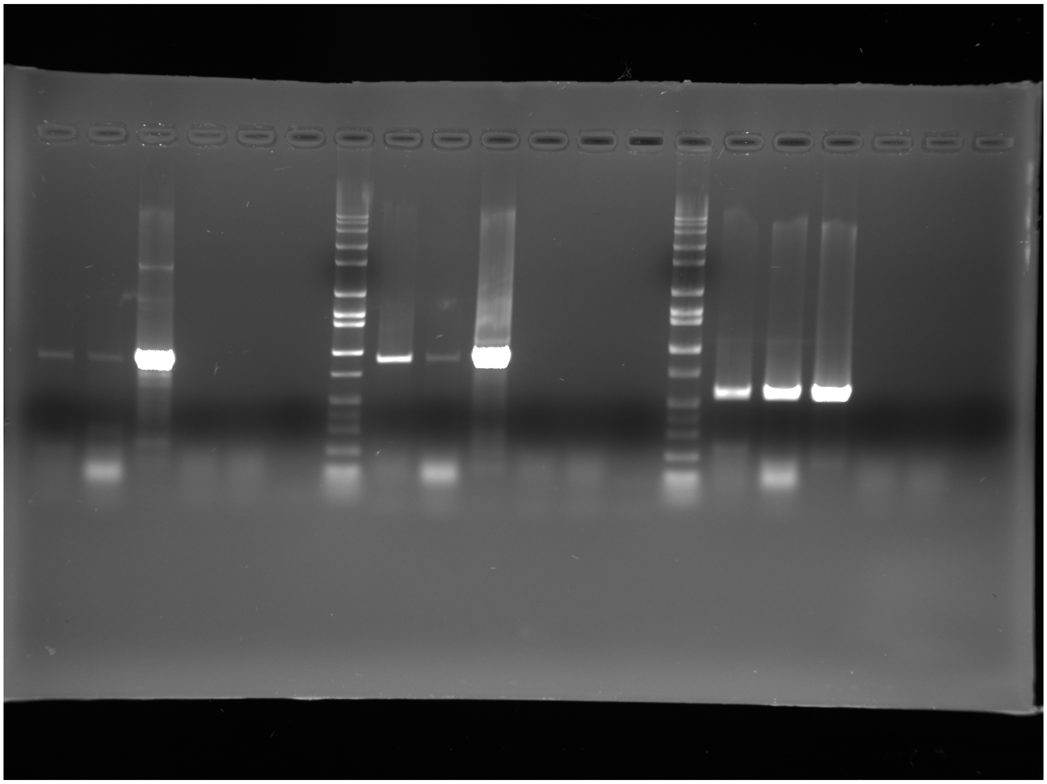

Figure S5. Full size agarose gel of RT-PCR analysis of *nlpD1* and *penA* expression. The inset shown in Figure 3(b) in the manuscript was derived from this picture of a 1.2 % agarose gel stained with ethidium bromide and imaged on a Bio-Rad ChemiDoc Imaging System. The resulting TIFF file was saved as a PDF and cropped to the desired size before being labeled in Powerpoint and inserted into composite Figure 3, which was assembled in Powerpoint. The darker area indicates loading dye.

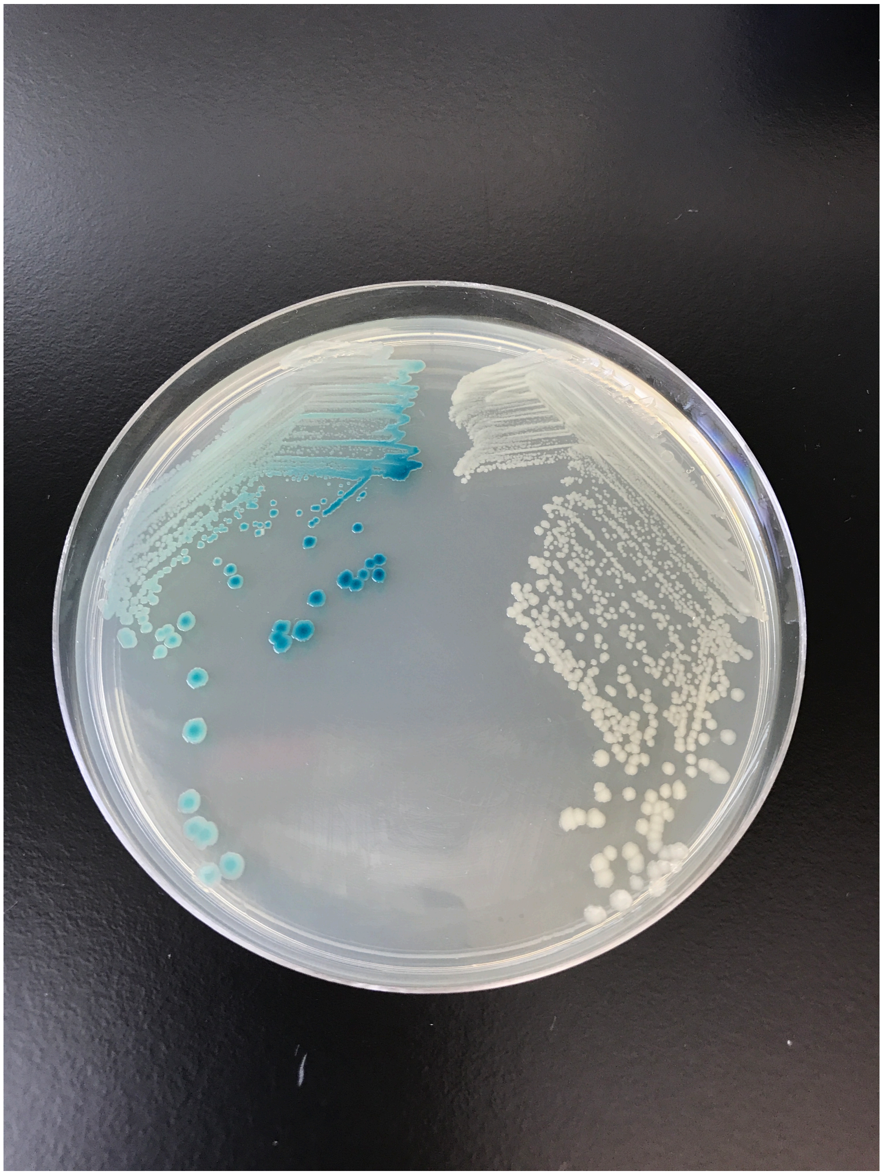

Figure S6. Full size picture of *E. coli* strains on an agar plate containing ß-galactosidase indicator. The inset shown in Figure 4(c) in the manuscript was derived from this picture of colonies on an LB agar plate with ß-galactosidase 5-bromo-4-chloro-3-indolyl-ß-D-galactopyranoside indicator substrate. The picture was taken with an iPhone 7 Plus. The picture JPEG file was saved as a PDF and cropped to the desired size before being labeled in Powerpoint and inserted into composite Figure 4, which was assembled in Powerpoint.

References

1. Kumar, A. *et al.* Expression of resistance-nodulation-cell division efflux pumps in commonly used *Burkholderia pseudomallei* strains and clinical isolates from Northern Australia. *Trans. Roy. Soc. Trop. Med. Hyg.* **102/S1**, S145-S151 (2008).
2. Winsor, G. L. *et al.* The Burkholderia Genome Database: facilitating flexible queries and comparative analyses. *Bioinformatics* **24**, 2803-2804, doi:DOI 10.1093/bioinformatics/btn524 (2008).
3. Yi, H. *et al.* Twelve positions in a beta-lactamase that can expand its substrate spectrum with a single amino acid substitution. *PloS One* **7**, e37585, doi:10.1371/journal.pone.0037585 (2012).
4. Bugrysheva, J. V. *et al.* Antibiotic resistance markers in strain Bp1651 of *Burkholderia pseudomallei* Identified by genome sequence analysis. *Antimicrob. Agents Chemother.* doi:10.1128/AAC.00010-17 (2017).
5. Rholl, D. A. *et al.* Molecular investigations of PenA-mediated beta-lactam resistance in *Burkholderia pseudomallei*. *Front. Microbiol.* **2**, 139, doi:10.3389/fmicb.2011.00139 (2011).
6. Randall, L. B., Dobos, K., Papp-Wallace, K. M., Bonomo, R. A. & Schweizer, H. P. Membrane bound PenA beta-lactamase of *Burkholderia pseudomallei*. *Antimicrob. Agents Chemother.* **60**, 1509-1514, doi:10.1128/AAC.02444-15 (2016).
7. Ferrandez, Y. & Condemine, G. Novel mechanism of outer membrane targeting of proteins in Gram-negative bacteria. *Mol. Microbiol.* **69**, 1349-1357, doi:10.1111/j.1365-2958.2008.06366.x (2008).
8. Viarre, V. *et al.* HxcQ liposecretin is self-piloted to the outer membrane by its N-terminal lipid anchor. *J. Biol. Chem.* **284**, 33815-33823, doi:10.1074/jbc.M109.065938 (2009).
9. Tsang, M. J., Yakhnina, A. A. & Bernhardt, T. G. NlpD links cell wall remodeling and outer membrane invagination during cytokinesis in *Escherichia coli*. *PLoS Genet.* **13**, e1006888, doi:10.1371/journal.pgen.1006888 (2017).
10. Uehara, T., Parzych, K. R., Dinh, T. & Bernhardt, T. G. Daughter cell separation is controlled by cytokinetic ring-activated cell wall hydrolysis. *EMBO J.* **29**, 1412-1422, doi:10.1038/emboj.2010.36 (2010).
